# Correlations Between Trace Elements in Selected Locations of the Human Brain in Individuals with Alcohol Use Disorder

**DOI:** 10.3390/molecules25020359

**Published:** 2020-01-15

**Authors:** Cezary Grochowski, Magdalena Szukała, Jakub Litak, Agnieszka Budny, Jędrzej Proch, Dariusz Majerek, Eliza Blicharska, Przemysław Niedzielski

**Affiliations:** 1 Department of Anatomy, Medical University of Lublin, Jaczewskiego 4, 20-090 Lublin, Poland; medszukala@op.pl (M.S.); aguskabudny@gmail.com (A.B.); 2 Department of Neurosurgery and Pediatric Neurosurgery, Medical University of Lublin, Jaczewskiego 8, 20-954 Lublin, Poland; jakub.litak@gmail.com; 3 Faculty of Chemistry, Department of Analytical Chemistry, Adam Mickiewicz University in Poznań, 89B Umultowska Street, 61-614 Poznan, Poland; jed.proch@gmail.com (J.P.); pnied@amu.edu.pl (P.N.); 4 Department of Applied Mathematics, University of Technology, Nadbystrzycka 38D, 20-618 Lublin, Poland; d.majerek@pollub.pl; 5 Department of Analytical Chemistry, Medical University of Lublin, Chodźki 4a, 20-093 Lublin, Poland; bayrena@o2.pl

**Keywords:** trace elements, correlation, brain, alcohol, alcohol use disorder

## Abstract

Trace element distribution varies in different locations of the human brain. Several elements were found to cause various negative effects, such as neurodegeneration. In this paper, we analyzed the interactions between seven trace elements: zinc (Zn), selenium (Se), manganese (Mg), iron (Fe), copper (Cu), chromium (Cr) and cobalt (Co) in individuals with alcohol use disorder (AUD) and individuals without (control group). Brain tissue samples from 31 individuals with AUD and 31 control subjects were harvested. Inductively coupled plasma optical emission spectrometry was used for trace element determination. In the control group, there were several positive correlations between Cr, Cu, Fe and Mn. In the AUD group, positive correlations between Co and Cr, Cu, Fe, Mn, Zn were found. The majority of correlations between Zn and other elements are positive. In the studied group, Mn had strong positive correlations with Co, Cr, Cu and Fe. The strongest positive correlation found between average element concentration was between Cu and Cr. The knowledge of kinetics and metabolism of trace elements as well as the impact of alcohol on these processes is essential for understanding the pathological processes and functioning of human brain tissue.

## 1. Introduction

Trace element distribution varies in different types of human tissue [1,2,3], as well as in different locations of the human brain. Several elements were found to have negative properties [4], inducing oxidative stress processes, and thus neurodegeneration [5,6,7]. The homeostasis of biometals is well regulated in healthy human brains and improper function of these systems may cause dysfunction of physiological brain processes [8]. The connection between improper trace element distribution and neurodegenerative diseases such as Alzheimer’s is well described in the literature [6]. Chronic copper, zinc and iron exposure was found to alter the processes of disease-associated proteins such as amyloid beta, amyloid precursor protein and tau protein.

Alcohol consumption is an important risk factor for disability, illness [9,10] and increased mortality [11]. People who regularly drink large amounts of alcohol, which can be defined as more than 40 g of pure alcohol per day by men and 20 g of pure alcohol per day by women are particularly vulnerable [12]. Not only is alcohol a source of trace elements itself, but chronic consumption of ethanol is a cause of malabsorption, malnutrition and is associated with vitamin and trace element deficiencies [13]. The correlations between trace elements in the human brain are still poorly described. In this paper, we analyzed the interactions between seven trace elements: zinc (Zn), selenium (Se), manganese (Mg), iron (Fe), copper (Cu), chromium (Cr) and cobalt (Co) in individuals with alcohol use disorder (AUD) and individuals without (control group).

## 2. Results

We discussed correlations that showed a strong or moderate association (*R* ≥ 0.7) presented in Figure 1 and Table 1 in more detail. Among the studied relationships, many correlations were found in different areas of the brain—the results are presented in Figure 2, Figure 3, Figure 4, Figure 5, Figure 6, Figure 7, Figure 8, Figure 9, Figure 10 and Figure 11.

In the Co–Al pair of elements, there was a slight positive correlation in the frontal cortex region, the caudate nucleus, the thalamus region and the cingular cortex region.

For the Co and Cr pair in the group exposed to alcohol, positive correlations were found for the following locations: frontal cortex, dorsal anterior cingular cortex, the head of the caudate nucleus and the frontal part of the thalamus. For this pair of elements in the control group, a moderate positive relationship was found in the frontal cortex region and a weak negative correlation was found in the foot of the hippocampus.

In the Co–Fe pair, a positive association was observed in the alcohol use disorder group in all studied regions, except the foot of the hippocampus and the frontal part of the insula region. In contrast, in the control group, for the nucleus accumbens, superior longitudinal fasciculus and frontal part of the insula region, a moderate negative correlation was found. The strongest negative correlation was in the frontal part of the insula.

For the pair of elements Co–Mn in the alcohol use disorder group, positive correlations were found in most of the studied structures, with the exception of the inferior longitudinal fasciculus, frontal part of the insula and foot of the hippocampus. A statistically significant negative relationship was observed in the group not exposed to the alcohol factor only for the frontal part of the insula structure.

In the Cr–Cu pair, a moderately positive relationship was found in 10 studied areas. In the control group, a similar relationship was observed in a few areas.

Between Cu and Fe, moderate and strong relationships were found in the examined areas of the brain in both groups included in the study.

In case of Cu–Mn, as for Zn–Mn, high correlation coefficients were observed in all studied areas, indicating a strong positive relationship. In the control group, such dependence is also observed, but slightly weaker.

Positive correlations of Cr and Mn were observed in both groups.

In the Cu–Zn pair, a positive correlation was found in the alcohol use disorder group in all studied regions. In the control group, the relationship was not statistically significant in most of the studied areas.

Between Fe and Mn, positive correlations were observed in all of the analyzed locations in both groups, however they were slightly stronger in the alcohol use disorder group.

In the group with alcohol use disorder for the studied Fe–Zn pair of elements, strong positive monotone relationships were found in all examined brain structures. The relationship was strongest in the frontal part of the insula.

The Mn–Zn pair of elements had high positive correlations in all tested areas of the group exposed to the alcohol factor. In the control group, a moderate positive association was found in the frontal cortex and superior longitudinal fasciculus region.

Correlation matrices comparisons by Schott test were performed (Table 2). Test statistics and *p*-values for particular regions are listed below. All comparisons showed significant differences in correlation matrices between groups.

## 3. Discussion

In this paper, several pairs of trace elements were presented which significantly statistically correlated with each other. Given that there are plenty of complex transport mechanisms for each element, it is problematic to discuss the kinetic interactions between these trace elements. However, further research is necessary regarding distribution and mutual correlation of trace elements [12,14,15,16,17].

There are approximately 7% of individuals throughout the world over 18 years of age who are considered to be habitual consumers of alcohol. Undoubtedly, alcoholic beverages are the source of different trace elements. Data presented by Darret et al. show that beer is a source of Cr and Se and wine contains large amounts of Al, Cr, Ni, Zn and Mg [18].

In the study assessing Cr and Se relationships in chicken brains, it was found that the group exposed to selenium and chromium had a lower concentration of chromium in tissues than the group exposed only to chromium. A clear negative correlation was observed in the pair of elements Cr and Se [19]. This does not correspond with the results obtained in this study. In the alcohol use disorder group, no significant relationship between these elements was found. On the other hand, in the control group, a moderate positive correlation was observed in most of the studied areas.

Research conducted by Rahil-Khazen et al. reports a positive correlation of many element pairs in the soft tissues of healthy patients (*n* = 30) such as the brain, liver, cortex and spleen. Most dependencies were found in the renal cortex and medulla (e.g., Fe–Co, Cu–Mn, Zn–Mn, Cu–Se and Zn–Cu). There was a strong positive relationship in the Fe–Co pair of trace elements in the brain [20]. On the contrary, in this study, a moderate negative relationship between Fe and Co in tissues from the nucleus accumbens, frontal part of the insula, anterior cingular cortex, and superior longitudinal fasciculus regions was found in the control group. In the alcohol use disorder group, a moderate positive correlation was found for most of the studied areas.

The literature describes a significant positive correlation between Cu, Fe, Zn and Mn in different human organs, which was consistent with the results obtained in this study [21]. Another study performed by Rahil-Khazen et al. reports that Zn–Fe had a negative correlation in soft tissues such as the brain, liver, kidney cortex and spleen, which is opposite to the results found in this study in patients with alcohol use disorder [22]. Moreover, several studies have shown that ethanol increases the permeability of the blood–brain barrier [23,24] as well as the blood–cerebrospinal fluid barrier [25]. Kornhuber et al. suggest that the permeability of these barriers is increased not only due to the dosage of consumed alcohol, but also by chronic intake.

The majority of correlations between elements in this study are positive and have bigger strengths among the group with alcohol use disorder compared to the control group, which may suggest a dysfunction within the blood–brain barrier in those individuals. Moreover, it is well known that disturbed homeostasis of elements such as Al [26], Fe [27], Zn and Cu [28] as well as alcohol itself induces oxidative stress processes, which increase the permeability of the blood–brain barrier. Moreover, it causes malfunction of endothelial cells [24], which are partially responsible for transportation within the blood–brain barrier [29]. Improper function of the blood–brain barrier may promote the accumulation of trace elements within brain tissue, which are known to cause neurodegeneration [30,31].

## 4. Materials and Methods

### 4.1. Population

Brain tissue samples were taken from individuals who underwent autopsy in the Department of Forensic Medicine, University of Lublin, Lublin, Poland. The authors harvested tissue samples from 31 people (8 female and 23 male) suffering from alcoholic use disorder. The inclusion criteria included no history of mental disorders, alcohol abuse reported in medical history as well as alcohol level over 2 per mil confirmed in blood at the time of the section. Moreover, thirty-one control subjects (Table 3) underwent tissue harvesting with inclusion criteria of macroscopically unaltered brain tissue, lack of documented neurodegenerative disease and alcoholic disorder history and blood alcohol level 0 per mil, confirmed at the time of the section. The study was approved by the Local Ethical Committee (Medical University of Lublin, Poland, approval no. KE-0254/2018) and the tissue collection was approved by the prosecutor’s office. The research has been carried out in accordance with The Code of Ethics of the World Medical Association, Declaration of Helsinki for experiments involving humans.

### 4.2. Procedure of Tissue Collection

Qualified pathologists collected the samples in accordance with the analytical protocol. Suprapure nitric acid solution (5% (*v*/*v*)) was used to decontaminate all of the materials that were in contact with the tissue samples. Moreover, ultrapure water (Milli-Q, Millipore, Raleigh, NC, USA; resistivity: 18.2 MΩ·cm) was used to thoroughly wash the tools.

The brain was removed from the cranium and washed with ultrapure water. Plastic tweezers were used to remove the meninges and the brain was rinsed with ultrapure water in order to reduce the probability of sample contamination with cerebrospinal fluid or blood. Disinfected plastic knives were used to collect tissue samples (approximately 0.5 g) from ten anatomical brain structures: frontal cortex (Broadmann area no. 11, PFC), dorsal anterior cingular cortex (Broadmann area no. 32, ACC), post central gyrus (Broadmann area no. 1, PCG), the foot of the hippocampus (HPC), frontal part of the thalamus (FTH), nucleus accumbens (NAC), frontal part of the insula (INS), the head of the caudate nucleus (CA), superior longitudinal fasciculus (SLF), and inferior longitudinal fasciculus (ILF). The selection of brain regions was based on their functional properties (memory, aggression, addiction, higher cognitive functions, emotion). Tissue samples were thoroughly rinsed with deionized water, drained on sterile blotting paper and weighed afterwards. All samples were put into sterile polypropylene containers (Bionovo, Legnica, Poland) and initially decayed with the use of 2 mL of 65% suprapure nitric acid. There was limited mass loss of the samples, which were digested directly after sampling without preliminary drying. In the last stage of the experiment, each sample was quantitatively transferred to closed Teflon containers and digested at 180 °C utilizing the microwave digestion system Mars 6 (CEM Corporation, Matthews, NC, USA). After digestion, samples were diluted with water to meet a total volume of 10.0 mL using scaled test-tubes.

### 4.3. Analytical Procedure

The inductively coupled plasma optical emission spectrometer Agilent 5110 ICP-OES (Agilent, Santa Clara, CA, USA) was employed for trace element determination. The synchronous vertical dual view (SVDV) of the plasma was accomplished by using dichronic spectral combiner (DSC) technology. This allows simultaneous axial and radial view analysis. In doing so, radio frequency (RF) power was 1.2 kW, nebulizer gas flow was 0.7 L min^−1^, auxiliary gas flow was 1.0 L min^−1^, plasma gas flow was 12.0 L min^−1^, charge coupled device (CCD) temperature was −40 °C, viewing height for radial plasma observation was 8 mm, and accusation time was 5 s. The analysis was repeated three times. The method has been validated using a wide organic matrix in several certified materials. The obtained results are given in the Supplementary Materials. Validation data for the determination of elements by this method are presented in Table 4. Due to the fact that certified reference materials (CRMs) of brain tissue are not available, the analytical method was additionally assessed by the standard additions method. ICP commercial analytical standards (Romil, Cambridge, UK) were used both for calibration and standard additions. Detection limits (DL) were determined through 3-sigma criteria and were on the level of 0.01 (mg/kg) wet weight (*w*/*w*) for all elements determined. The uncertainty for the complete analytical process (including sample preparation) was at the level of 20%. Traceability was assessed by comparison with reference materials. A recovery of 80–120% was considered acceptable for all the elements determined.

### 4.4. Statistical Analysis

The correlations between concentrations of individual elements in the brain were analyzed in terms of their magnitude and direction. Correlation and covariance analyses were performed. Since elemental concentrations in different areas may have an abnormal distribution, Spearman′s coefficient was used to test the correlation. Spearman’s correlation coefficients were calculated for each brain region divided into control and alcohol use disorder groups. *p*-values were also included in figures (numbers in brackets). Intervals for correlations in pairs of elements in two studied groups are presented in Table 3. The differences between matrices of correlations were tested with the Schott test, which is a generalization of the Box M test for homogeneity of covariance matrices. This generalization is robust for non-normality. All variables were scaled before testing, so covariance matrices turned to correlation matrices [14]. The significance level (*p*-value) adopted in this work is 0.05. All statistical calculations and visualizations were done in R environment (R Core Team 2019 R Foundation for Statistical Computing, Vienna, Austria) with extra packages such as ggplot2, dplyr, purrr, and ggcorrplot.

## 5. Conclusions

The results of this study showed a great number of correlations between different trace elements in the human brain. Statistically significant alterations were found between individuals with alcohol use disorder and the control group. More correlations were observed in the AUD group and they were stronger than those found in the controls. The knowledge of kinetics and metabolism of trace elements, as well as the impact of alcohol to this process, is essential for understanding the pathological processes and functioning of human brain tissue.

## Figures and Tables

**Figure 1:**
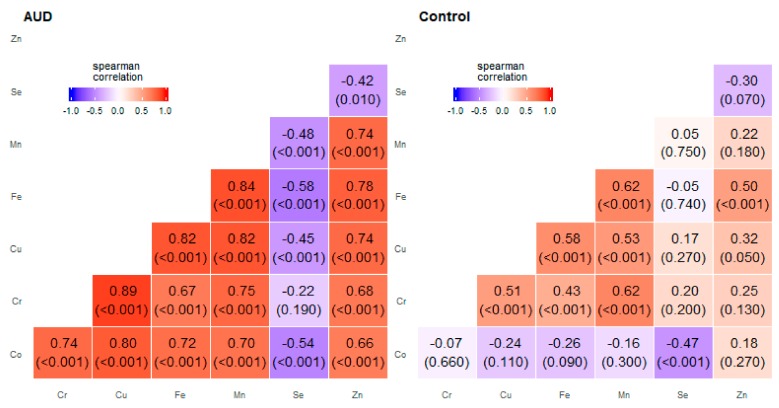
Correlation matrix of average brain trace element concentrations in the alcohol use disorder group and control group. The strongest relationships between average concentrations of trace elements are marked in circles.

**Figure 2:**
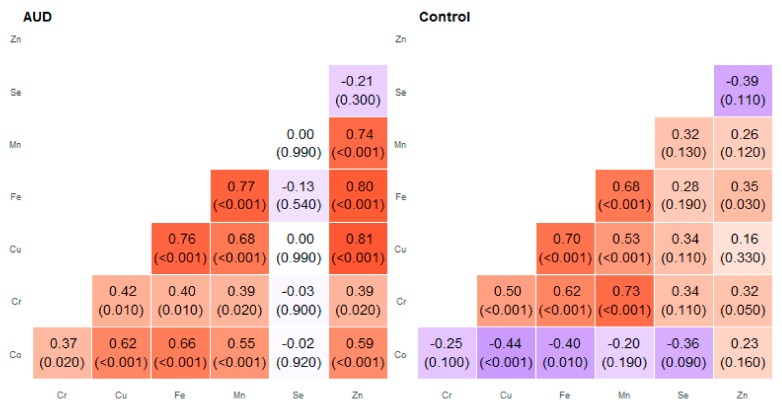
Correlation matrix of Spearman coefficient charts in the anterior cingular cortex region in the alcohol use disorder and control groups.

**Figure 3:**
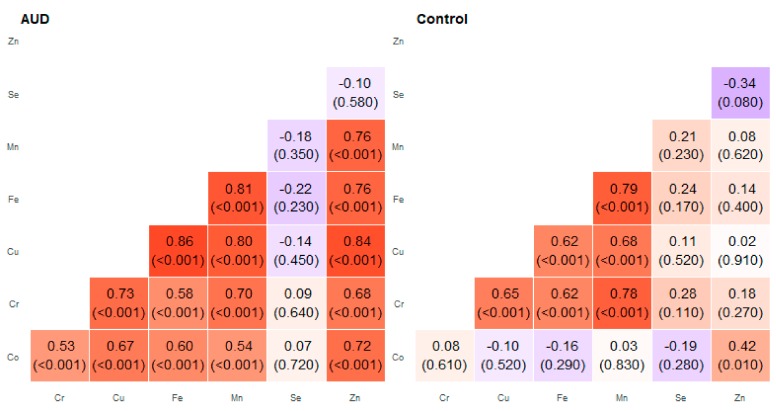
Correlation matrix of Spearman coefficient charts in the head of the caudate nucleus region in the alcohol use disorder and control groups.

**Figure 4:**
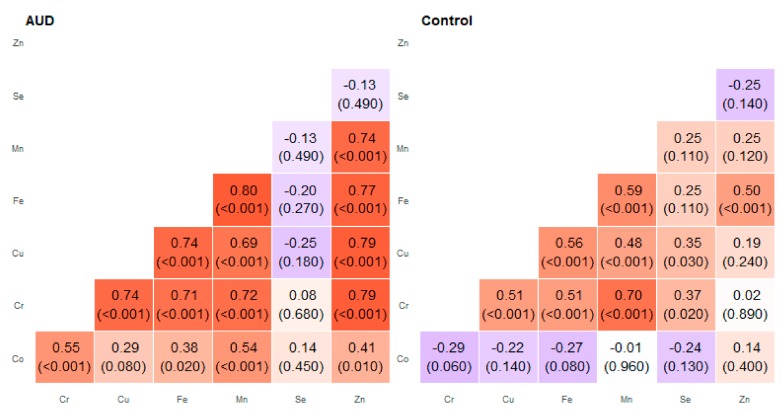
Correlation matrix of Spearman coefficient charts in the frontal part of the thalamus region in the alcohol use disorder and control groups.

**Figure 5:**
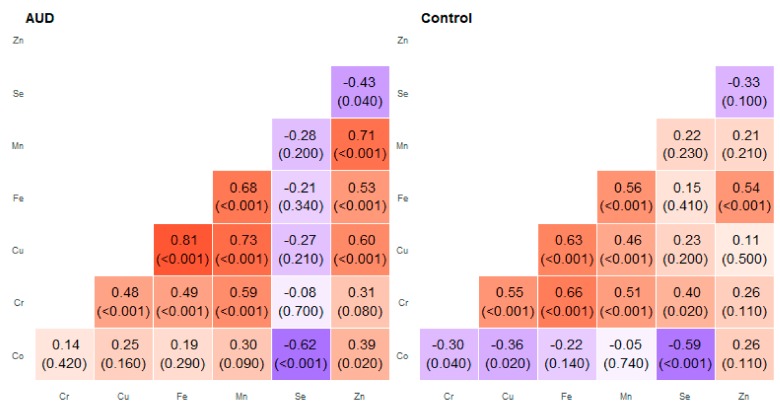
Correlation matrix of Spearman coefficient charts in the foot of the hippocampus region in the alcohol use disorder and control groups.

**Figure 6:**
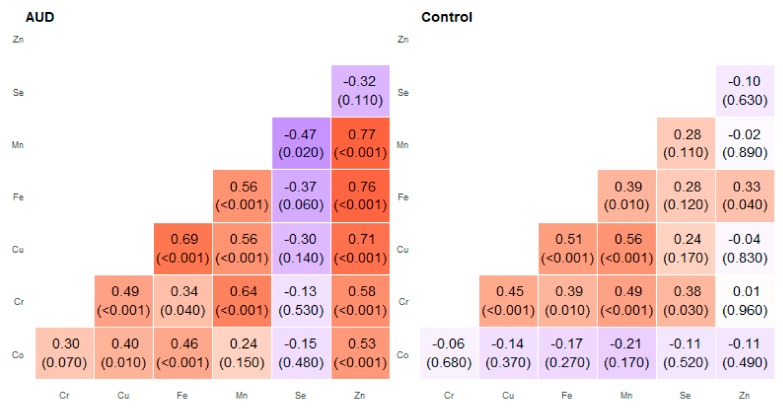
Correlation matrix of Spearman coefficient charts in the inferior longitudinal fasciculus region in the alcohol use disorder and control groups.

**Figure 7:**
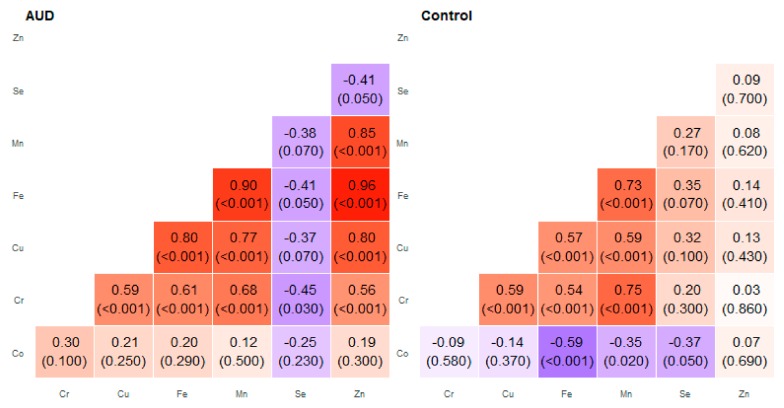
Correlation matrix of Spearman coefficient charts in the frontal part of the insula region in the alcohol use disorder and control groups.

**Figure 8:**
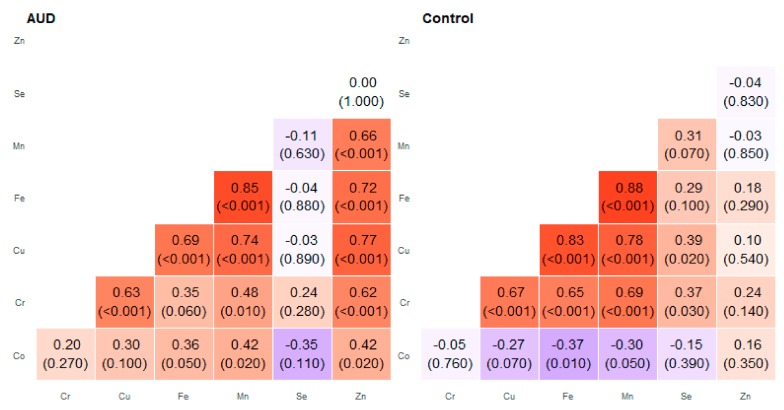
Correlation matrix of Spearman coefficient charts in the nucleus accumbens region in the alcohol use disorder and control groups.

**Figure 9:**
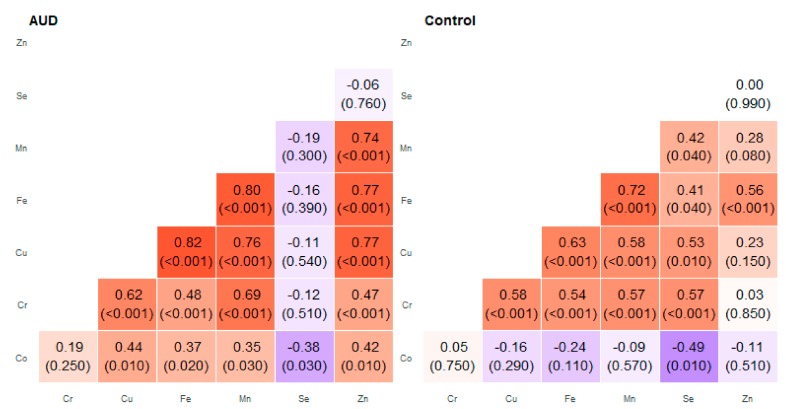
Correlation matrix of Spearman coefficient charts in the post central gyrus region in the alcohol use disorder and control groups.

**Figure 10:**
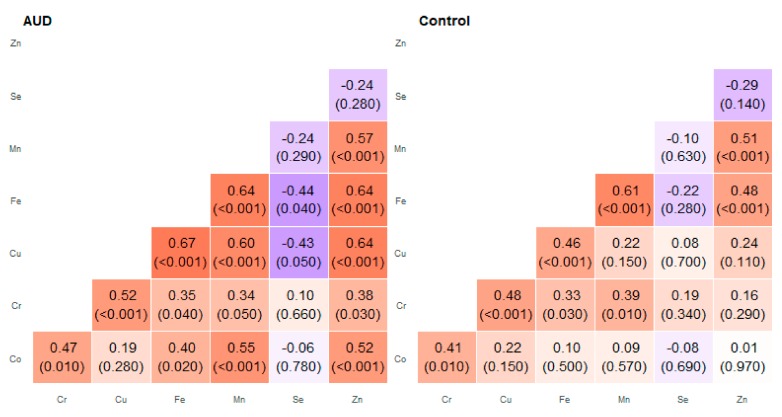
Correlation matrix of Spearman coefficient charts in the frontal cortex region in the alcohol use disorder and control groups.

**Figure 11:**
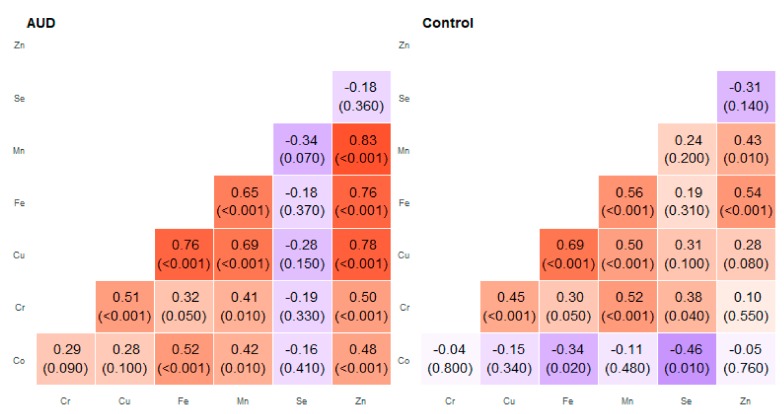
Correlation matrix of Spearman coefficient charts in the superior longitudinal fasciculus region in the alcohol use disorder and control groups.

**Table 1:** Intervals for correlations in selected pairs of elements in the two studied groups.

Pair of Trace Elements	Alcohol Use Disorder Group		Control Group	
	Lower Bound	Upper Bound	Lower Bound	Upper Bound
Co–Cr	0.03	0.74	−0.30	0.41
Co–Cu	0.21	0.62	−0.44	0.22
Co–Fe	0.19	0.72	−0.59	0.62
Co–Mn	0.12	0.69	−0.35	0.09
Cr–Cu	0.42	0.74	0.45	0.67
Cr–Mn	0.34	0.72	0.39	0.78
Cu–Fe	0.67	0.86	0.46	0.83
Cu–Mn	0.56	0.8	0.22	0.78
Cu–Zn	0.52	0.84	−0.10	0.32
Fe–Mn	0.45	0.90	0.39	0.88
Fe–Zn	0.26	0.97	0.10	0.56
Mn–Zn	0.57	0.85	−0.03	0.51

**Table 2:** Tests for equality of correlation coefficient matrices by Schott method.

Region	Statistic	*p*-value
Dorsal anterior cingular cortex	56.91	<0.001
The head of the caudate nucleus	26.58	<0.001
Frontal part of the thalamus	41.46	<0.001
The foot of the hippocampus	27.39	<0.001
Inferior longitudinal fasciculus	40.44	<0.001
Frontal part of the insula	37.78	<0.001
Nucleus accumbens	38.32	<0.001
Post central gyrus	38.04	<0.001
Frontal cortex	42.20	<0.001
Superior longitudinal fasciculus	29.71	<0.001
Total (average value)	65.09	<0.001

**Table 3:** Demographic characteristics of analyzed subjects. AUD = alcohol use disorder, SD = standard deviation.

Parameter	Control Group	AUD Group	*p*
Age (mean ± SD)	50.3 ± 19.1	47.9 ± 13.3	0.41
Sex (n, %)	Women: 8 (26%) Men: 23 (74%)	Women: 10 (32%) Men: 21 (68%)	0.58
Weight (mean ± SD)	82.0 ± 20.9	81.4 ± 25.4	0.64
BMI (kg/m^2^, mean ± SD)	28.5 ± 7.3	26.5 ± 7.4	0.65

**Table 4:** Validation data for elements determination by inductively coupled plasma optical emission spectroscopy (ICP-OES).

	Wavelength	DL	Range	Uncertainty
	nm	mg L^−1^	mg L^−1^	%
Co	238,892	0.00029	DL-20	1.6
Cr	267,716	0.00033	DL-20	4.0
Cu	327,395	0.00027	DL-20	9.6
Fe	238,204	0.00084	DL-100	1.6
Mn	257,610	0.00021	DL-20	1.8
Se	196,026	0.0011	DL-10	16.4
Zn	213,857	0.00022	DL-20	3.5

## Supplementary Materials

The following are available online at https://www.mdpi.com/1420-3049/25/2/359/s1, Table S1: Descriptive statistics. Statistics were calculated for each element by tested areas and groups (for data before logarithmic transformation). Table S2: Results of certified reference materials analysis
