# Supplementary material for: Correlations Between Trace Elements in Selected Locations of the Human Brain in Individuals with Alcohol Use Disorder
Source: Molecules. 2020 Jan 15;25(2):359. doi: 10.3390/molecules25020359 (PMC7024425; doi:10.3390/molecules25020359)
Supplement: Supplementary file 1 [file molecules-25-00359-s001.pdf]

Suplementary Material

Correlations Between Trace Elements in Selected Locations of the Human Brain in Individuals with Alcohol Use Disorder

Cezary Grochowski [1,2,*], Magdalena Szukała [1], Jakub Litak [2], Agnieszka Budny [1], Jędrzej Proch [3], Dariusz Majerek [5], Eliza Blicharska [4] and Przemysław Niedzielski [3]

[1] Department of Anatomy, Medical University of Lublin, Jaczewskiego 4, 20-090 Lublin, Poland; medszukala@op.pl (M.S.); aguskabudny@gmail.com (A.B.)

[2] Department of Neurosurgery and Pediatric Neurosurgery, Medical University of Lublin, Jaczewskiego 8, 20-954 Lublin, Poland; jakub.litak@gmail.com

[3] Faculty of Chemistry, Department of Analytical Chemistry, Adam Mickiewicz University in Poznań, 89B Umultowska Street,61-614 Poznan, Poland; jed.proch@gmail.com (J.P.); pnied@amu.edu.pl (P.N.)

[4] Department of Analytical Chemistry, Medical University of Lublin, Chodźki 4a, 20-093 Lublin, Poland; bayrena@o2.pl

[5] Department of Applied Mathematics, University of Technology, Nadbystrzycka 38D, 20-618 Lublin, Poland; d.majerek@pollub.pl

* Correspondence: cezary.grochowski@o2.pl

Table 1. Descriptive statistics. Statistics were calculated for each element by tested areas and groups (for data before logarithmic transformation).

ACC - dorsal anterior Cingular cortex (Broadmann area no. 32),
CA - the head of the caudate nucleus,
FTH - frontal part of the thalamus,
HPC the foot of the hippocampus,
ILF – the inferior longitudinal fasciculus,
INS – the frontal part of the insula,
NAC - the nucleus accumbens,
PCG - post central gyrus (Broadmann area no. 1),
PFC – the frontal cortex (Broadmann area no. 11),
SLF - superior longitudinal fasciculus,
Total - Average brain trace element concentations (average of all presented concentations),
K- control group
AUD – group with alcohol use disorder

Element	Region	Group	min	mean	median	max	sd
Co	ACC	K	0.018	0.329	0.304	0.918	0.168
Co	ACC	AUD	0.010	0.316	0.300	1.420	0.256
Co	CA	K	0.010	0.171	0.168	0.410	0.073
Co	CA	AUD	0.010	0.121	0.118	0.466	0.098
Co	FTH	K	0.010	0.174	0.148	0.511	0.090
Co	FTH	AUD	0.010	0.146	0.148	0.381	0.099
Co	HPC	K	0.016	0.264	0.256	0.528	0.126
Co	HPC	AUD	0.039	0.248	0.200	0.651	0.146
Co	ILF	K	0.010	0.266	0.236	0.709	0.134
Co	ILF	AUD	0.010	0.260	0.268	0.542	0.153
Co	INS	K	0.106	0.272	0.227	0.638	0.140
Co	INS	AUD	0.036	0.207	0.179	0.718	0.135
Co	NAC	K	0.010	0.197	0.188	0.367	0.077
Co	NAC	AUD	0.010	0.159	0.142	0.466	0.117
Co	PCG	K	0.010	0.289	0.287	0.620	0.151
Co	PCG	AUD	0.010	0.221	0.198	0.673	0.155
Co	PFC	K	0.090	0.308	0.262	0.631	0.143
Co	PFC	AUD	0.028	0.313	0.267	0.885	0.192
Co	SLF	K	0.010	0.257	0.227	0.596	0.132

Co	SLF	AUD	0.010	0.244	0.238	0.501	0.155
Co	Total	K	0.017	0.247	0.260	0.407	0.084
Co	Total	AUD	0.023	0.203	0.203	0.415	0.102

**Element**	**Region**	**Group**	**Min**	**Mean**	**Median**	**Max**	**Sd**
Cr	ACC	K	0.029	0.606	0.139	5.203	1.315
Cr	ACC	AUD	0.061	0.143	0.132	0.400	0.058
Cr	CA	K	0.050	0.514	0.140	4.724	1.111
Cr	CA	AUD	0.042	0.119	0.118	0.192	0.037
Cr	FTH	K	0.092	0.789	0.164	7.234	1.825
Cr	FTH	AUD	0.000	0.130	0.135	0.243	0.058
Cr	HPC	K	0.052	0.579	0.135	5.679	1.301
Cr	HPC	AUD	0.060	0.141	0.130	0.586	0.086
Cr	ILF	K	0.084	0.575	0.221	3.918	1.036
Cr	ILF	AUD	0.060	0.197	0.202	0.318	0.054
Cr	INS	K	0.010	0.817	0.157	7.296	1.923
Cr	INS	AUD	0.072	0.148	0.129	0.353	0.055
Cr	NAC	K	0.012	0.626	0.167	5.377	1.332
Cr	NAC	AUD	0.092	0.157	0.143	0.334	0.060
Cr	PCG	K	0.010	0.575	0.151	4.634	1.234
Cr	PCG	AUD	0.069	0.138	0.124	0.272	0.051
Cr	PFC	K	0.018	0.167	0.141	0.557	0.105
Cr	PFC	AUD	0.010	0.118	0.122	0.202	0.036
Cr	SLF	K	0.087	1.104	0.235	21.606	3.425
Cr	SLF	AUD	0.000	0.212	0.219	0.359	0.065
Cr	Total	K	0.066	0.600	0.164	5.256	1.287
Cr	Total	AUD	0.067	0.138	0.139	0.218	0.038

**Element**	**Region**	**Group**	**Min**	**Mean**	**Median**	**Max**	**Sd**
Cu	ACC	K	1.653	13.092	4.693	95.011	24.910
Cu	ACC	AUD	0.746	3.925	3.229	9.362	1.940
Cu	CA	K	2.531	13.519	6.322	103.142	22.447

Cu	CA	AUD	1.845	5.179	4.758	12.065	2.502
Cu	FTH	K	1.599	26.471	4.367	289.239	67.504
Cu	FTH	AUD	0.288	3.781	3.533	11.811	2.184
Cu	HPC	K	1.142	9.729	3.689	76.526	18.160
Cu	HPC	AUD	0.000	3.143	2.658	12.941	2.178
Cu	ILF	K	1.514	11.648	4.589	81.397	21.082
Cu	ILF	AUD	1.512	3.567	3.454	7.693	1.418
Cu	INS	K	1.695	36.698	5.200	552.858	104.206
Cu	INS	AUD	0.964	4.366	3.738	10.043	2.072
Cu	NAC	K	3.169	16.063	7.520	115.411	26.347
Cu	NAC	AUD	2.202	6.217	5.748	12.918	2.687
Cu	PCG	K	0.010	11.211	5.299	66.962	18.101
Cu	PCG	AUD	1.238	4.345	3.947	12.033	2.241
Cu	PFC	K	1.282	4.497	4.258	18.619	2.506
Cu	PFC	AUD	1.113	3.544	3.578	7.917	1.460
Cu	SLF	K	1.752	23.509	5.148	220.753	55.198
Cu	SLF	AUD	0.000	3.840	3.494	8.320	1.598
Cu	Total	K	1.805	13.326	5.091	108.462	25.019
Cu	Total	AUD	1.255	3.836	3.335	9.339	1.789

**Element**	**Region**	**Group**	**Min**	**Mean**	**Median**	**Max**	**Sd**
Fe	ACC	K	8.543	174.372	69.050	1394.160	334.061
Fe	ACC	AUD	17.903	45.101	42.990	91.111	18.343
Fe	CA	K	27.145	379.249	256.813	1751.535	426.725
Fe	CA	AUD	21.784	172.798	137.523	402.695	107.643
Fe	FTH	K	17.525	272.350	133.078	1990.494	432.241
Fe	FTH	AUD	5.391	99.384	80.225	227.739	53.527
Fe	HPC	K	8.623	215.959	77.805	1591.086	407.799
Fe	HPC	AUD	11.395	59.139	58.949	186.287	31.417
Fe	ILF	K	10.895	276.799	121.480	2020.169	482.923
Fe	ILF	AUD	31.683	72.213	68.834	126.508	28.428
Fe	INS	K	17.029	267.168	96.854	1913.870	504.777

Fe	INS	AUD	23.930	83.364	61.621	578.783	97.567
Fe	NAC	K	26.965	492.265	357.984	1836.781	437.194
Fe	NAC	AUD	59.966	248.525	219.727	613.819	159.395
Fe	PCG	K	0.014	219.458	112.331	1461.148	354.355
Fe	PCG	AUD	24.352	73.620	71.160	132.441	31.725
Fe	PFC	K	13.141	83.918	70.716	315.762	61.174
Fe	PFC	AUD	18.574	54.715	44.424	195.749	32.033
Fe	SLF	K	8.990	266.169	99.085	2003.161	489.608
Fe	SLF	AUD	31.150	62.791	54.289	121.335	25.464
Fe	Total	K	19.264	248.008	135.620	1772.171	363.335
Fe	Total	AUD	16.466	88.655	71.498	191.182	47.095

**Element**	**Region**	**Group**	**Min**	**Mean**	**Median**	**Max**	**Sd**
Mn	ACC	K	0.103	105.900	0.352	1118.100	302.234
Mn	ACC	AUD	0.171	0.311	0.296	0.566	0.103
Mn	CA	K	0.204	83.897	0.694	770.251	238.170
Mn	CA	AUD	0.145	0.570	0.552	1.157	0.245
Mn	FTH	K	0.150	93.691	0.577	1030.368	269.262
Mn	FTH	AUD	0.030	0.494	0.479	0.959	0.204
Mn	HPC	K	0.112	86.628	0.460	856.286	247.276
Mn	HPC	AUD	0.214	0.412	0.384	1.169	0.176
Mn	ILF	K	0.126	78.139	0.463	936.485	226.312
Mn	ILF	AUD	0.195	0.457	0.439	1.288	0.217
Mn	INS	K	0.124	70.545	0.371	743.097	200.783
Mn	INS	AUD	0.118	0.427	0.304	1.970	0.390
Mn	NAC	K	0.209	76.402	0.776	735.512	214.487
Mn	NAC	AUD	0.265	0.692	0.642	1.593	0.370
Mn	PCG	K	0.010	97.264	0.377	1139.303	287.188
Mn	PCG	AUD	0.125	0.397	0.297	2.657	0.444
Mn	PFC	K	0.075	0.461	0.314	2.757	0.510
Mn	PFC	AUD	0.143	0.273	0.268	0.530	0.086
Mn	SLF	K	0.169	98.705	0.593	1116.068	284.665

Mn	SLF	AUD	0.209	0.514	0.511	1.205	0.211
Mn	Total	K	0.139	77.366	0.497	884.211	234.668
Mn	Total	AUD	0.167	0.413	0.377	0.883	0.172

**Element**	**Region**	**Group**	**Min**	**Mean**	**Median**	**Max**	**Sd**
Se	ACC	K	0.038	3.225	0.635	14.437	5.331
Se	ACC	AUD	0.000	0.510	0.426	1.485	0.371
Se	CA	K	0.018	3.173	0.474	21.590	6.757
Se	CA	AUD	0.010	0.418	0.427	1.101	0.255
Se	FTH	K	0.000	2.374	0.508	18.356	5.279
Se	FTH	AUD	0.029	0.442	0.334	1.712	0.359
Se	HPC	K	0.043	2.793	0.478	20.355	5.747
Se	HPC	AUD	0.017	0.477	0.432	1.413	0.354
Se	ILF	K	0.013	1.771	0.428	10.836	3.289
Se	ILF	AUD	0.031	0.577	0.604	1.233	0.327
Se	INS	K	0.021	3.325	0.504	19.184	6.400
Se	INS	AUD	0.027	0.465	0.334	1.772	0.415
Se	NAC	K	0.020	1.946	0.408	12.019	3.798
Se	NAC	AUD	0.069	0.459	0.418	1.130	0.262
Se	PCG	K	0.043	3.395	0.492	17.406	5.975
Se	PCG	AUD	0.027	0.537	0.444	1.351	0.403
Se	PFC	K	0.040	0.551	0.489	1.613	0.428
Se	PFC	AUD	0.024	0.428	0.401	1.046	0.313
Se	SLF	K	0.021	3.054	0.614	21.265	5.942
Se	SLF	AUD	0.028	0.473	0.444	0.917	0.284
Se	Total	K	0.077	1.772	0.263	18.378	4.392
Se	Total	AUD	0.090	0.323	0.281	0.810	0.179

**Element**	**Region**	**Group**	**Min**	**Mean**	**Median**	**Max**	**Sd**
Zn	ACC	K	6.848	18.192	18.970	24.789	4.652
Zn	ACC	AUD	4.206	16.370	14.741	31.496	5.908
Zn	CA	K	7.171	18.763	20.008	25.158	4.714

Zn	CA	AUD	6.308	15.464	15.121	26.732	5.700
Zn	FTH	K	6.503	17.735	18.613	26.621	4.402
Zn	FTH	AUD	1.191	16.189	14.947	79.978	12.207
Zn	HPC	K	7.616	21.957	22.992	36.935	5.755
Zn	HPC	AUD	5.080	23.636	17.823	208.636	33.741
Zn	ILF	K	4.376	14.044	14.988	20.100	3.983
Zn	ILF	AUD	4.739	11.661	11.969	20.047	3.837
Zn	INS	K	8.923	19.649	20.278	31.350	5.019
Zn	INS	AUD	8.325	27.992	17.436	379.986	64.405
Zn	NAC	K	6.516	22.334	21.135	119.424	16.814
Zn	NAC	AUD	7.320	16.548	16.066	29.138	6.059
Zn	PCG	K	5.264	18.924	16.687	101.000	14.313
Zn	PCG	AUD	4.598	13.811	12.839	25.550	5.192
Zn	PFC	K	7.246	24.217	18.558	120.045	22.130
Zn	PFC	AUD	4.959	15.388	15.865	26.876	5.030
Zn	SLF	K	3.899	11.764	12.377	20.246	3.038
Zn	SLF	AUD	4.030	11.869	9.606	66.044	9.694
Zn	Total	K	3.138	17.834	18.721	31.001	5.117
Zn	Total	AUD	3.460	15.205	13.971	43.383	7.784

2. Table S2. Results of certified reference materials analysis

	CRM CS-M-1			CRM NCSDC			CRM S-1			CRM 2709			CRM 405			CRM 667		
	certified mg kg^{-1}	determined mg kg^{-1}	recovery %	certified mg kg^{-1}	determined mg kg^{-1}	recovery %	certified mg kg^{-1}	determined mg kg^{-1}	recovery %	certified mg kg^{-1}	determined mg kg^{-1}	recovery %	certified mg kg^{-1}	determined mg kg^{-1}	recovery %	certified mg kg^{-1}	determined mg kg^{-1}	recovery %
**Co**	x	0,11	x	0,41	0,38	93	3,9	3,4	87	12,8	12,6	98	13,7	14,0	102	23	21,0	91
**Cr**	x	0,48	x	2,6	2,1	81	38	40	105	130	143	110	84	75	89	178	169	95
**Cu**	x	7,1	x	6,6	6,1	92	6,3	5,9	94	x	21	x	47,7	43	90	60	62	103
**Fe**	x	104	x	1070	943	88	9880	9553	97	33600	29459	88	37400	32655	87	44800	40890	91
**Mn**	x	12	x	61	56	92	266	286	107	529	461	87	495	398	80	920	723	79
**Se**	1,37	1,5	106	x	0,21	x	x	<0,01	x	1,5	1,20	80	0,44	0,50	114	1,59	1,5	94
**Zn**	60,9	62	102	55	58	105	35	38	109	103	98	95	279	235	84	175	167	95
